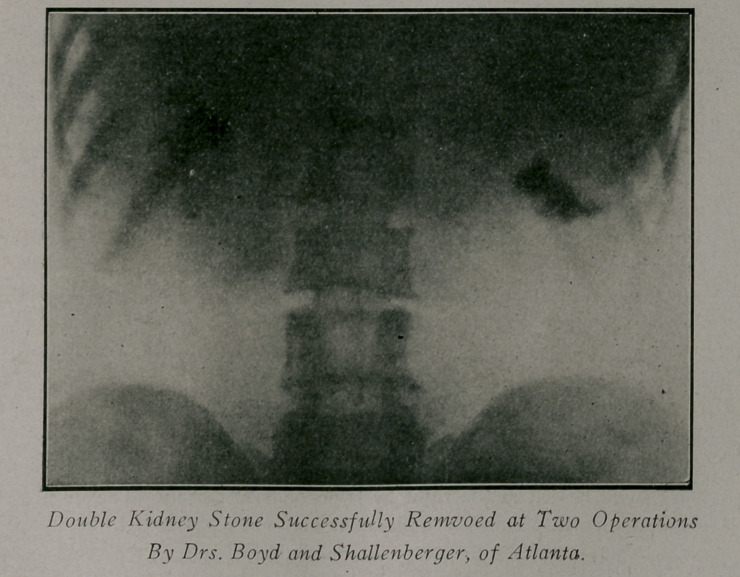# The Value of the Roentgen Rays in Diagnosis of Kidney Disease

**Published:** 1912-08

**Authors:** John S. Derr

**Affiliations:** Atlanta, Ga.


### Journal-Record of Medicine

Successor to Atlanta Medical and Surgical Journal, Established 1855
and Southern Medical Record, Established 1870.

**OWNED BY THE ATLANTA MEDICAL JOURNAL CO.**

**Published Monthly**

*Official Organ Fulton County Medical Society, State Examining Board, Presbyterian Hospital, Atlanta, Birmingham and Atlantic Railroad Surgeons' Association, Chattahoochee Valley Medical and Surgical Association, Etc.*

EDGAR G. BALLENGER, M. D., Editor.
BERNARD WOLFF, M. D., Supervising Editor.
A. W. STIRLING, M. D., C. M., D. P. H., J. S. HURT, B. Ph., M. D.
GEO. M. NILES, M. D., W. J. LOVE, M. D., (Ala.); Associate Editors.
E. W. ALLEN, Business Manager.

COLLABORATORS

Dr. W. F. WESTMORLAND, General Surgery.
F. W. McRAE, M. D., Abdominal Surgery.
H. F. HARRIS, M. D., Pathology and Bacteriology.
E. B. BLOCK, M. D., Diseases of the Nervous System.
MICHAEL HOKE, M. D., Orthopedic Surgery.
CYRUS W. STRICKLER, M. D., Legal Medicine and Medical Legislation.
E. C. DAVIS, A. B., M. D., Obstetrics.
E. G. JONES, A. B., M. D., Gynecology.
R. T. DORSEY, Jr., B. S. M. D., Medicine.
L. M. GAINES, A. B., M. D., Internal Medicine.
GEO. C. MIZELL, M. D., Diseases of the Stomach and Intestines.
L. B. CLARKE, M. D., Pediatrics.
EDGAR PAULIN, M. D., Opsonic Medicine.
THEODORE TOEPEL, M. D., Mechano Therapy.
R. R. DALY, M. D., Medical Society.
A. W. STIRLING, M. D., etc., Diseases of the Eye, Ear, Nose and Throat.
BERNARD WOLFF, M. D., Diseases of the Skin.
E. G. BALLENGER, M. D., Diseases of the Genito-Urinary Organs.

VOL. LIX. AUGUST 1912 NO. 5

### THE VALUE OF THE ROENTGEN RAYS IN DIAGNOSIS OF KIDNEY DISEASE.

BY DR. JOHN S. DERR, ATLANTA, GA.

Although a comparatively new science, the Roentgen Rays have already, in the seventeen years since its discovery, contributed immensely to the advancement of both medicine and surgery. Of all the achievements of medicine, this can be considered only less great than the discovery of Vaccination, by Jenner; Antiseptic Surgery, by Lister, and the germ theory of disease by Pasteur. Of its many uses it has a very important field in the diagnosis of kidney disease, being used, of course, in conjunction with the other methods of examination.

Indeed, no obscure case of a kidney lesion will admit of a final diagnosis without all the information which the X-ray examination can give. Its value depends largely on the following factors: A good machine, a tube of the proper vacuum, a skillful technique and the proper development and interpretation of the plate after it is obtained. The preparation of the patient may also be an important factor in the result, as masses of feces and bubbles of gas in the intestine destroy the clearness of the X-ray negative, and the former may even simulate a stone. The intestinal tract should be thoroughly cleared with castor oil and food should be sparingly taken of before the examination. This is made with the patient in the dorsal position and the lumbar curve brought down to the table as closely as possible, the plate being placed beneath, and extending from the 10th rib downward to the iliac crest. On very stout subjects the abdominal binder will be found useful to reduce the thickness of the abdomen. In all cases the compression diaphragm of Albers-Schonberg with hemispherical aluminum window is of great advantage to push the intestines out of the path of the rays.

The cone should be tilted at such an angle as to throw the line of focus upward beneath the costal border, if possible; the degree of the angle to be suggested by the anatomy of the patient. By this procedure the kidney is penetrated diagonally and its shadow is brought out with the greatest possible distinctness, the distortion produced being very trifling.

Unless the patient is small, I find it best to make a separate focus and exposure for each kidney.

For examining the lower ureter for stone, the focus of the tube should be directed downward at a considerable angle in order to project the shadow through the pelvic canal and onto the plate. My apparatus is a 4 k. w. Wate & Bartless Transformer, running at 110 volt, direct current. The character of Roentgen tube should be such that 40 to 50 millamperes of current can be passed, using the full power. The softer the tube the better, provided it has the necessary penetration. This tends to bring out the greatest amount of detail in the soft structures, such as the pdros muscle, liver and spleen. Too hard a tube and too long an exposure may penetrate the calculus.

The time exposure varies according to the thickness of the subject from two to five seconds by time switch. Such a technique renders it possible to make as many exposures as may be desired, without the slightest danger of injury to the patient.

In the early days of the X-ray, it was not uncommon to expose for 30 or 40 minutes in efforts to penetrate the abdomen,

*The Shadow of a Soft or Paste Stone in the Same Situation from Which a Formed Stone Had Been Removed about a Year Previous. Operation by Dr. Floyd McRae, of Atlanta.*

and a case is on record of a woman who, after three such exposures developed a sloughing wound from which she died. The dread of the X-ray burn still persists in the minds of many who are not familiar with the advances made in modern technique.

The exposure is made while the patient holds his breath, either in expiration or inspiration; both have their advantages in particular cases, though, as a rule, the former is the best, as the kidneys move downward with the diaphragm and the costal margins are more elevated.

Having discussed the technique, we next come to diagnosis. What can the X-ray tell us about the kidney? The Fleuroscope, though of little value in the direct examination of the organ, gives very valuable information as to the gravity of the patient's condition in chronic kidney disease, by examination of the lungs. Williams, of Boston, has discovered that in Nephritis there is a darkening of the bases of the lungs, due to edema and that this can be detected before physical signs are present.

The following conditions may be demonstrated on the negative: The position, size and shape of the organ, and whether or not one is absent; atrophy and hypertrophy, by comparison of the two sides, and in cases where palpation is impossible on account of the obesity of the subject, these conditions may be demonstrated, provided dense abdominal adhesions do not interfere. When these are very extensive a clear-cut outline of the kidney may be impossible to show. With a clear abdomen, however, any irregularity of outline caused by a tumor mass could be readily detected.

Of course, it would be very difficult to determine, from the Skiagraph alone, whether a regular enlargement is due to simple hypertrophy or disease unless a stone shadow were present, but the functional test in this case would clear up the matter.

The difference in density between the normal kidney tissue and a diseased portion due to malignancy or Tuberculosis might possibly be brought out with ideal conditions, but I have never had the opportunity of seeing it demonstrated. It has been reported that a Tuberculosis process, extending into the pelvis of

*Collargol Injection Showing the Course of the Ureter and Dilatation of the Kidney Pelvis and Calices with Tilting of the Kidney. Injection done by Dr. E. G. Ballenger, of Atlanta.*

the organ, will absorb a solution of colorgol injected through the ureter, and this ought to give a good idea of the extent of the disease. This method of injecting the ureter with a non-irritant silver solution, preferably 15% colorgol, was originated by Dr. Garrity, of Baltimore, and is of great value in determining the size and conformation of the kidney pelvis and culices and showing up the course of the ureter in which it reveals the presence of kinks and points of compression or twisting. It also shows the relation which a calculus bears to the pelvis of the organ, especially in cases where the kidney shadow cannot be brought out. To determine whether or not a shadow in the course of the lower ureter is due to a calculus or flebolith in one of the iliac veins a stilette passed into the ureter is simpler and more serviceable than the injection. There are three straights or narrowings of the lumen in the course of the ureter in any of which a small calculus may find a lodgment. The first is just below the pelvis of the ureter; the next is just above sacro-iliac junction, and the third just above the bladder. The last is the most frequent of lodgment.

It is in the diagnosis of renal and uretrical calculus that the X-ray finds its greatest efficiency in kidney diagnosis. The great frequency of this condition and the numerous signs and symptoms which give excellent grounds for suspicion where no stone exists, combine to make the debt of the medical profession very great to a means of diagnosis so accurate.

Were it the only thing that Roentgen's great discovery ever did for medicine, that alone ought to be sufficient to make his name immortal. The first diagnosis of stone by the X-ray was made by Dr. Mackintyre, of Gasgow, in 1896.

Permit me to quote some authorities cited by Kassabian, of Philadelphia, who died a martyr to the new science, the dangerous nature of which was not realized until it was too late. Dr. Chas. Leonard, one of the greatest authorities in America, says: The absolute negative and positive diagnosis of calculus nephritis and ureteritis can be made with an error of less than 3%. In 320 cases examined, calculi were found in 97 and in 47 of these symptoms demanded operative interference and in all but one the accuracy of a negative diagnosis was proven and no calculus was found.

Kummel and Rumpel report a series of 18 cases diagnosed positively by the X-rays, all of which were subsequently operated on and the stone extracted. From their work they have formulated the following conclusions:

*Stone in the Pelvis of the Ureter. Operated on by Drs. Ballenger and Elder, of Atlanta.*

1. The exact diagnosis of kidney stones is made only by means of the Roentgen procedure.

2. The presence of a kidney stone, whether located in the kidney substance, in the calices or in the ureter will be demonstrated on the plate in every case, by a proper application of the Roentgen method.

3rd. The negative results of the Roentgen method, after repeated attempts, allow of the exclusion of the calculus.

4. The demonstrtation of a stone shadow upon the Roentgen plate is not dependent upon the size and chemical composition of the calculus, but singly and alone on the technique of the Roentgen operator.

5. The X-rays, properly used, will detect a stone in any individual, no matter how thick.

Walsh, of London, in his Roentgen Rays in Medical Work, quotes Hutchison:

Renal calculi, however small, should be operated on as soon as they are diagnosed. This danger to the kidney structure and to the patient's life bears no relation to their size.

So far as my own experience has gone, nothing could be more disappointing than to attempt to draw definite conclusions from the signs, symptoms and history of the case. The patient may have typical and repeated attacks of violent pain in the lumbar region which may or may not radiate down into the groin and testicle, and the urine reported to contain pus, blood and albumen; he may even have a history of having passed a stone at some time or other. The diagnosis is made, and we feel as sure of the presence of a stone as if it had already been removed. The X-ray plate then confronts us with two beautifully clear kidney shadows, perfectly free from anything which could be interpreted as a stone.

On the other hand, the symptoms may have been very indefinite. A tendency to headache, an occasional ache in the lumbar region and the urine showing a trace of pus or albumen. We make the examination with skepticism and are surprised to find a beautiful and unmistakable shadow in the "correct situations" or framed in the kidney outline. I am convinced that tne most pronounced symptoms and signs, including hoematuria, can be produced by the kinking of the ureter or a passive congestion of the organ, due to prolapse with pressure on the renal vessels. Of course, both conditions may exist at the same time and if operative interference is indicated, it is of great importance to ascer-

*The Upper Shadow is a Formed Stone. Those Below are Masses of "Paste" or "Sand" in a Fistulous Tract which Opened in the Back Just Above the Crest of the Ileum. Operation by Dr. Floyd McRae, of Atlanta.*

tain the presence of a stone, if one exists. Then, again, stone in the kidney or ureter has been frequently diagnosed as appendicitis and ovarian trouble. Beginning spinal caries is said to have symptoms closely resembling those of renal calculus. The detection of the stone depends on its casting a shadow more dense than the soft structures which surround it, the kidney itself being more dense than the abdominal muscles and the intestines. This makes it imperative that the plate should be of such a character as to cast shadows of structures less dense than the least dense stone

To aid in determining the position of the stone, a French authority has formulated a rule that if the stone shadow is 3 1-2 x to 4 1-2 c. m. from the midline of the spine, some part of it is in the kidney pelvis; if a greater distance, it was in the substance. This would be of value where the shadow of the kidney is indistinct. I have found it to be correct several times and wrong at least once. The density of the shadow cast by calculi varies according to their chemical compositions, as follows: the calcium oxolate gives the best shadow, next the phosphetic and lastly that composed of uric acid, and urates. Kassobian says that the latter is only slightly less opaque to the rays than compact bone. If this is true, there should be no difficulty in detecting a stone of this composition on a plate of the proper quality. As a matter of fact, the pure uric acid stone is extremely rare, though it frequently occurs mixed with phosphates and oxolates, which facilitate its detection. It is a remarkable fact that the law of Roentgen; namely, the more dense the object the deeper the resulting shadow, does not hold good in the case of calcula. The calcum oxolate having the lower sp. gr. and biliary calculi, the most permeable of all stones having the highest.

There are two possible sources of error in the diagnosis of stone which are beyond the power of Roentgen technique to eliminate; one of these is a question of interpretation. A stone shadow may be so small that the Radiologist hesitates to make a positive diagnosis. In a case of this kind the examination should be made repeatedly at intervals of several days. The smallest stone which I have seen reported diagnosed before operation was in the ureter and weighed one-fifth of a grain.

The other factor mentioned is not an error except in the sense that it may give rise to a discrepancy between the evidence of the X-ray plate and the surgical findings. I know no better name for this than the soft stone. A collection of stone forming material or paste which may be in the renal pelvis, in a calix or a pocket in the kidney substance, and which may be very difficult of a positive demonstration at operation. This material casts a shadow which I defy any radiologist to distinguish from that of a formed stone. It gives rise to the same symptoms and is of equally

*A Number of Large Stones Contained in the Shell of the Destroyed Kidney. Successfully Removed by Drs. McRae and Roberts, of Atlanta.*

great surgical importance, being simply a stone in embryo. I have only seen one case mentioned in literature—David Walsh, of England reports this case, where at operation an ill-formed mass was found at the bottom of an old scar and removed with a spoon. This, to my mind, greatly enhances the value of a negative diagnosis. But in cases of positive diagnosis, it makes the result so disappointing to all parties concerned that I shall never again permit a case to come to operation without a warning of the possibilities. I do not believe that any mass of scar tissue, however dense, will give the picture of a stone.

Out of a total number of 69 cases examined to date, the positive diagnosis of stone has been made 18 times—two of these in the same patient about a year apart. In five a colorgol injection of the ureter and kidney pelvis was done. In three cases the examination was not satisfactorily completed because of the patient's failure to return. From one of these a stone was afterward removed. Of the 18 positive cases, two were in the lower ureter just above the bladder and in both cases stones were subsequently passed. Eleven cases were operated on, the remaining five have not yet come to operation. Of those operated on, definite formed stones were found in seven. In four of these the stones were multiple as indicated by the plate. One of these the stones were contained in a pocket in the ureter, and one was a double stone. In another the entire kidney was simply a mass of stones. There were two cases of pus kidney diagnosed before operation and found to be completely destroyed. In one of these the two small stones on which the diagnosis was made, were never found. Another case which showed two shadows was so far destroyed that the kidney had to be removed. In another diagnosis of double stone was made. One side was operated on and stone paste found in pelvis. Another showed a spot of putty-like substance to the searching needle in the situation indicated by the plate. Of the five cases injected with colorgol, a stone had already been diagnosed in one. Two showed definite kinks in the ureter, one of which also had abnormally large branching calieas; one had a dilated pelvis and one was a prolapse kidney. All four were confirmed by operation and symptoms cured. In the remaining 51 cases, which were pronounced negative for stone, so far as I am aware—in not a single ated on and stone paste found in the pelvis. Another showed a case has the diagnosis been proven incorrect.

Case 1.—Referred by Dr. McRae. Patient, male; age, 34; weight, 170 lbs. History of albuminuria and intermittent attacks of pain in the left side over a period of six years. The X-ray plate showed a large, irregular stone shadow which cut the 12th rib, and a smaller one below it. Both stones were removed at operation, with a good recovery. About a year later the patient again had symptoms and passed a small stone. The urine from he left side showed pus and blood. The X-Ray examination showed a perfectly clear-cut and unusually dense shadow, al-

*Stone in the Pelvis of the Kidney. Not Yet Come to Operation. Referred by Dr. E. G. Ballenger, of Atlanta.*

most the size of a hickory nut, in the same situation from which the first stone had been removed. The operation revealed the old scar and a putty-like resistance to the examining needle in the situation of the first stone.

Case 2.—Referred by Dr. Ballenger. Patient, male; age, about 30; weight 160. History of frequent attacks of kidney colic and haematuria since a boy of 14. Cystocopic examination showed a profuse flow of pus from the right ureter. The X-ray plate showed a shadow of the right kidney which was considerably enlarged and two small shadows in the lower pole which changed their relative position on three different plates. Diagnosis of pus kidney was made. This was confirmed at operation, but the two small shadows on which the diagnosis was made were never accounted for. Recovery very good.

Case 3.—Reported by Dr. Ballenger. Patient, male; aged 53; weight 150 lbs. History of pain in the right side for three or four years and at times the urine showed pus and blood. These attacks would clear up and the urine show only a small amount of albumen. The plates showed a stone of the size of a hazel nut in the pelvis of the right ureter and several smaller ones in the substance of the kidney; also a suspicious shadow in the inferior straight of the ureter. Several months later the kidney findings were confirmed by operation and the organ which was almost destroyed was removed. Recovery uneventful.

Case 4.—Referred by Dr. McRae. Patient, female; age about 35; weight 130 lbs. History of operation for abscess of the left kidney nine years before. Healing did not take place and a later operation showed the formation of a stone. Removal was followed by healing, but several months later colic returned and more stones were removed from a fistula in the back. The plate showed a number of well defined shadows scattered from the 12th rib to the crest of the ilium. The lower ones very far out. At operation the kidney was found to be destroyed and contained one well formed stone. The shadows in the fistulous tract were made up of a friable material or stone paste which has already been mentioned. Recovery good.

Case 5.—Referred by Dr. Boyd. Patient, male; age 30; weight, 120 lbs. History of pain beginning seven or eight years ago, with typical symptoms of renal colic in both sides. At this time he passed two stones, but none since. Catheterization showed pus from both ureters. The plates demonstrated stones in both kidneys which were removed at two different operations, with recovery.

Case 6.—Referred by Dr. Todd. Patient, female; aged 25; weight 154 lbs. History of pain in the left side, beginning two years ago. The attacks were frequent and the pain radiated downward into the groin and inner side of thigh. X-ray examination showed the left kidney and upper ureter to be clear, but in the course of the lower ureter just above the bladder shadow was what appeared to be a stone about the size and shape of a grape seed. A number of confirmatory plates were made. After several attacks of severe pain the patient finally passed a small

*Double Kidney Stone Successfully Remvoed at Two Operations By Drs. Boyd and Shallenberger, of Atlanta.*

mulberry calculus, corresponding in size and shape to the shadow on the plate.

Case 7.—Referred by Dr. E. C. Davis. Patient, male; aged 25; weight 140 lbs. History of repeated attacks of kidney colic in the left side, accompanied by haematuria. No stone passed. The plate demonstrated a shadow in the pelvis of the left kidney; also a less distinct one in the upper pole of the right. The left side was operated on and the pelvis was found to contain debris or stone paste.

Case 6.—Referred by Dr. Dorsey. Patient, male; age 43; weight 155 lbs. History of several attacks of pain in the region of the left kidney within a few months. Pain radiating to groins and penis. Urine contains pus and blood. The skiagraph showed the left kidney and upper ureter to be clear of stone. The bladder region showed a small, irregular shadow in the course of the ureter just above the bladder. Some time after, a small stone was passed.

Case 9.—Referred by Dr. Champion. Patient, male; age 26; weight 140. History of dull, aching pain in the left side for four years. Urine sometimes cloudy and sometimes clear. No blood passed that he knows of. Several X-ray examinations made at Johns-Hopkins about a year previous pronounced negative for stone. A plate including both kidneys showed a shadow in the left kidney region overlying the 12th rib. It was about the size of a filbert and appeared to be in the pelvis of the kidney. As a precauton against error, the patient was thoroughly purged and another exposure made which entirely confirmed the findings of the first. The stone was removed at operation.

Case 10.—Referred by Dr. E. G. Jones. Patient, male; age 52; weight 140. History of pain in right side four years previous, and passed a small stone two years later, with blood. No definite symptoms since that time until five weeks before, when severe pain came in the right side, which continued. A definite mass could be felt in the right by hypocondriac region. The urine was normal. The X-ray plate showed what appeared to be a mass of stones in the ureter about one-half inch above the crest of the ilium. Kidney shadow higher up and very large. Pus kidney diagnosed before operation. A large pus kidney was removed and a sack containing a number of stones was removed from what had been the ureter.

Case 11.—Dr. E. G. Jones (Contrast Case). Patient, aged 36; weight 140 lbs. History of pain in the right side, beginning two years before, accompanied by the formation of a swelling which has given more or less pain ever since. There was a violent attack four weeks previous, with pains radiating down into the testicles. Palpation showed a tender mass on the right side, three fingers below the rib margin. The kidney shadow was brought out clearly on the plate and free from stone, but a soft tumor could be defined below and internal to it. Operation revealed a multilocular cyst of the omentum.

Case 12.—Referred by Dr. Roberts. Patient, male; age 48; weight 212. History of having passed several small stones fifteen years before. Since then he has had numerous attacks of left sided colic and has passed another small stone. The urine has been constantly cloudy and there is a history of a bad malarial infection. Though the more definite attacks of colic have been referred to the left side, there has been a more or less constant feeling of uneasiness in the region of the right kidney. Ureteral catheterization showed the urine from the left kidney to be clear and a flow from the right to be cloudy. It was reported that the right kidney was doing one-third of the work of the left. The X-ray of the right kidney showed the entire organ to be apparently calcified and modular. The ureter to the bladder was clear. At operation the kidney was found to be little more than a shell containing a number of stones, some of which were very large.

355-56-57 Candler Annex.

## Figures and Tables

**Figure f1:**
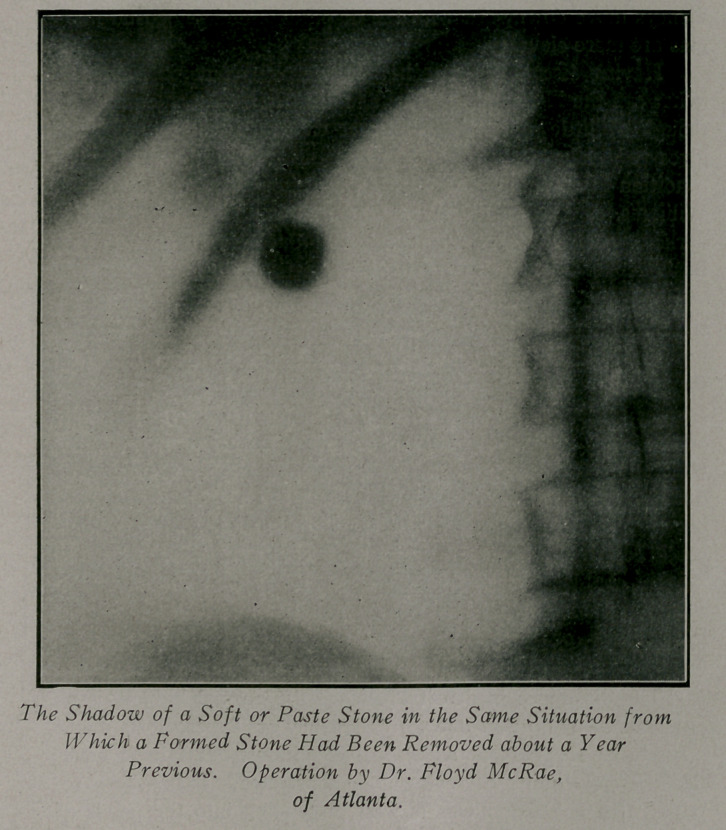


**Figure f2:**
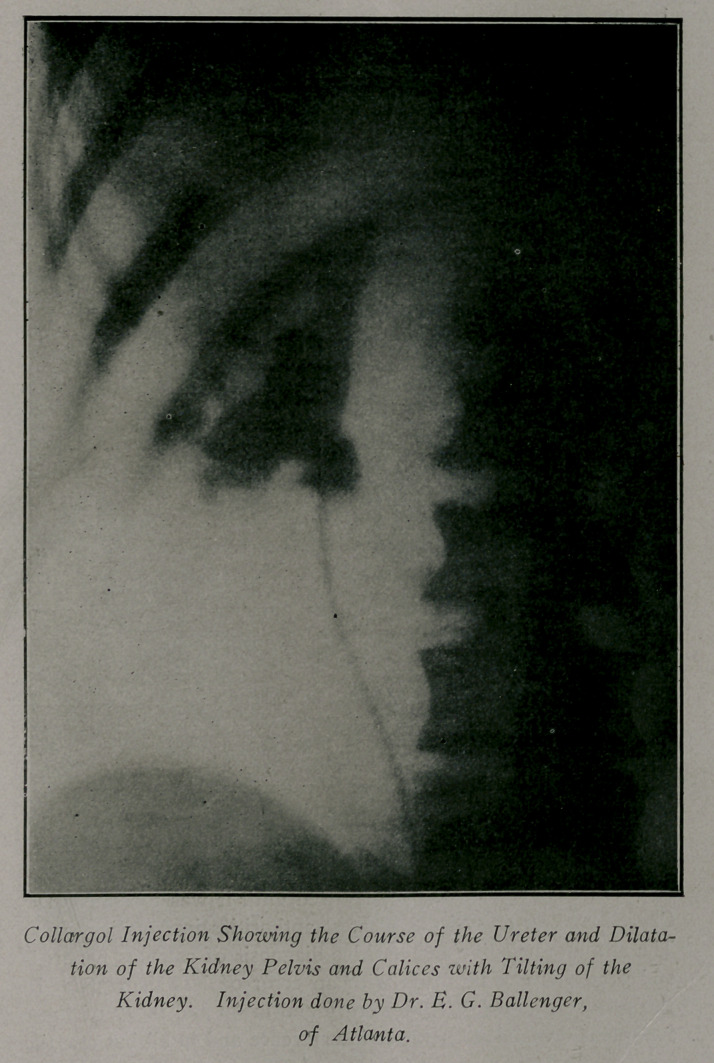


**Figure f3:**
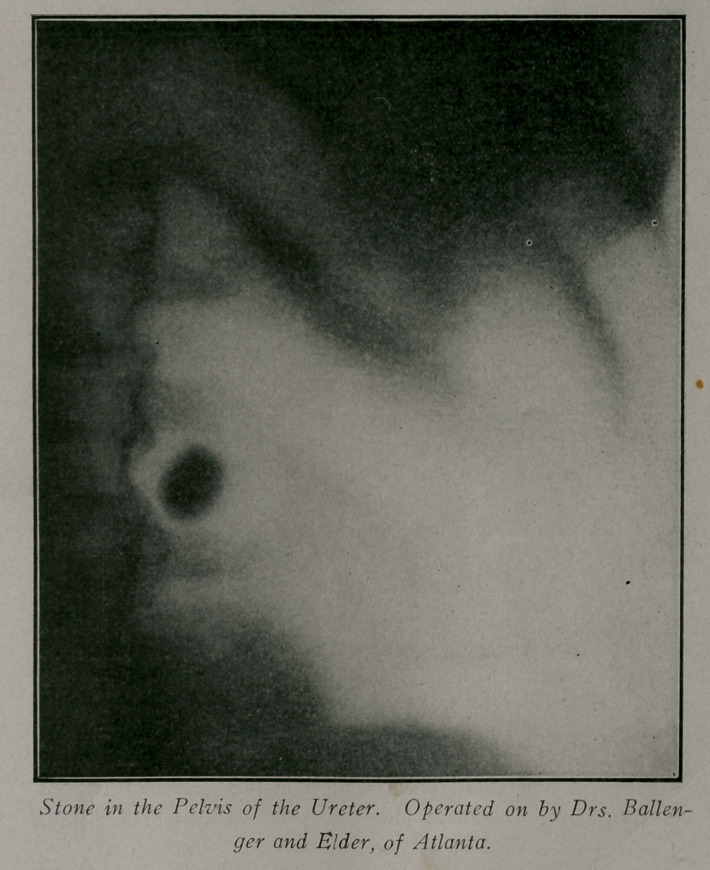


**Figure f4:**
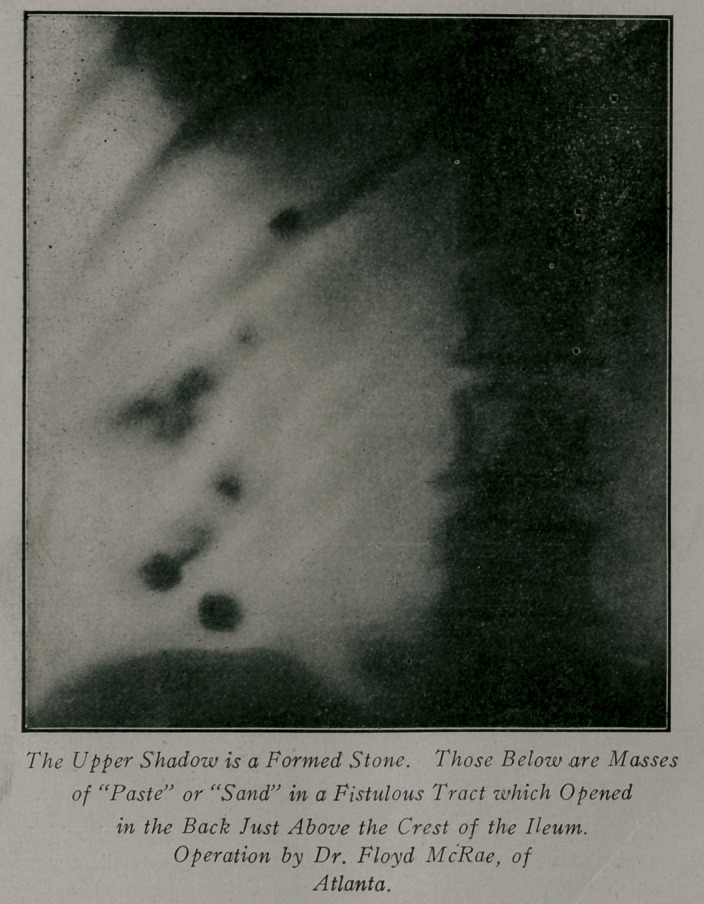


**Figure f5:**
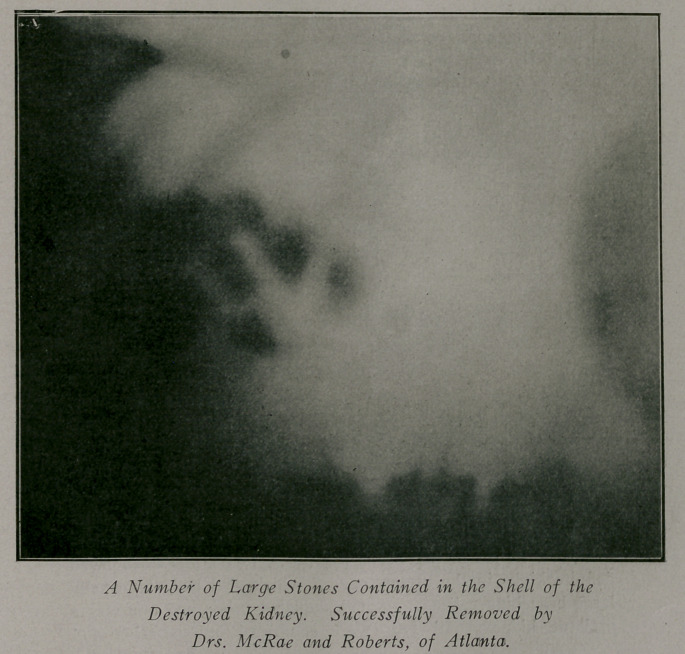


**Figure f6:**
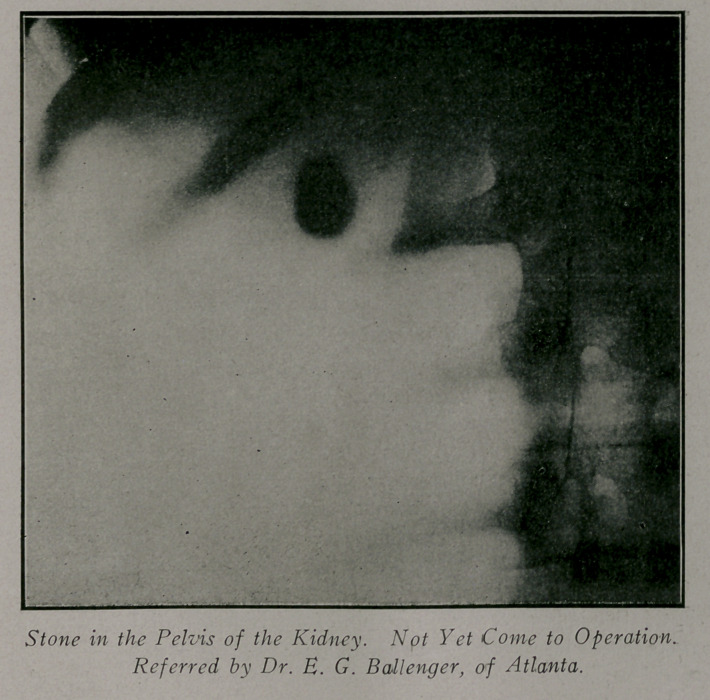


**Figure f7:**